# Reliability and validity of the Patient Health Questionnaire-4 scale and its subscales of depression and anxiety among US adults based on nativity

**DOI:** 10.1186/s12888-024-05665-8

**Published:** 2024-03-18

**Authors:** David Adzrago, Timothy J. Walker, Faustine Williams

**Affiliations:** 1 Division of Intramural Research, National Institute on Minority Health and Health Disparities, National Institutes of Health, 11545 Rockville Pike, 20852 Rockville, MD USA; 2 Center for Health Promotion and Prevention Research, The University of Texas School of Public Health, The University of Texas Health Science Center at Houston, Houston, TX USA

**Keywords:** Anxiety/depression, COVID-19 pandemic, Nativity, Patient health questionnaire, Psychometric properties, Mental health

## Abstract

**Background:**

The burdens of anxiety and depression symptoms have significantly increased in the general US population, especially during this COVID-19 epidemiological crisis. The first step in an effective treatment for anxiety and depression disorders is screening. The Patient Health Questionnaire-4 (PHQ-4, a 4-item measure of anxiety/depression) and its subscales (PHQ-2 [a 2-item measure of depression] and Generalized Anxiety Disorder [GAD-2, a 2-item measure of anxiety]) are brief but effective mass screening instruments for anxiety and depression symptoms in general populations. However, little to no study examined the psychometric properties (i.e., reliability and validity) of the PHQ-4 and its subscales (PHQ-2 and GAD-2) in the general US adult population or based on US nativity (i.e., foreign-born vs. the US-born). We evaluated the psychometric properties of the PHQ-4 and its subscales in US adults, as well as the psychometric equivalence of the PHQ-4 scale based on nativity.

**Methods:**

We conducted a cross-sectional survey of 5,140 adults aged ≥ 18 years. We examined the factorial validity and dimensionality of the PHQ-4 with confirmatory factor analysis (CFA). A multiple-group confirmatory factor analysis (MCFA) was used to evaluate the comparability of the PHQ-4 across nativity groups. Reliability indices were assessed. Also, the scales’ construct validities were assessed by examining the associations of both the PHQ-4 and its subscales’ scores with the sociodemographic characteristics and the 3-item UCLA Loneliness scale.

**Results:**

The internal consistencies were high for the PHQ-4 scale (α = 0.92) and its subscales of PHQ-2 (α = 0.86) and GAD-2 (α = 0.90). The CFA fit indices showed evidence for the two-factor structure of the PHQ-4. The two factors (i.e., anxiety and depression) were significantly correlated (*r* = 0.92). The MCFA demonstrated measurement invariance of the PHQ-4 across the nativity groups, but the model fits the data better in the foreign-born group. There were significant associations of the PHQ-4 scale and its subscales’ scores with the sociodemographic characteristics and the UCLA Loneliness scale (all *p* < 0.001).

**Conclusions:**

The PHQ-4 and its subscales are reliable and valid measures to screen anxiety and depression symptoms in the general US adult population, especially in foreign-born individuals during the COVID-19 pandemic.

## Background

Mental health disorders, including anxiety (i.e., feeling nervous or anxious and worried) and depression (i.e., mood disorder and anhedonia/lack of pleasure), are common in the U.S., yet they often go untreated [1–3]. In 2019, about 18.1% (40 million) and 6.7% (16.1 million) of U.S. adults experienced anxiety and depression disorders, respectively [1]. However, only 36.9% and 61.7% of these adults received treatments for anxiety and depression disorders, respectively [1]. These disorders have increased significantly, especially during the COVID-19 pandemic; between 2019 and 2020, anxiety (2019 = 18.1% vs. 2020 = 30.8%) and depression (2019 = 6.7% vs. 2020 = 23.5%) increased more than two-fold among U.S. adult population [1, 4]. Anxiety and depression are strong co-occurring disorders and have increased disability severity due to their comorbidity [5, 6]. For instance, at least half of individuals who experience a depression disorder also experience an anxiety disorder [1]. Anxiety and depression symptoms have also impacted COVID-19 vaccination, such that individuals with anxiety and depression symptoms are not willing to be vaccinated [7–9]. The burdens of anxiety and depression symptoms are concerning, especially during this COVID-19 epidemiological crisis.

Substantial evidence suggests the first step in an effective treatment for anxiety and depression disorders is screening [10–15]. Hence, there is a need for better screening for anxiety and depression in the general U.S. population. The detection of these disorders, especially their comorbidity, and their mass treatments in the general adult population requires a mass but brief screening instrument.

Anxiety and depression are commonly screened with a reliable and valid 4-item self-reported instrument called Patient Health Questionnaire-4 (PHQ-4) [12, 13]. The PHQ-4 measures both anxiety and depression symptoms. It consists of two subscales: PHQ-2 (a 2-item measure of depression) and Generalized Anxiety Disorder (GAD-2, a 2-item measure of anxiety) [12, 13]. The PHQ-4 is an ultra-brief screening instrument for the symptoms, which are not indicators of clinical diagnostic disorders but indicators for further assessments by mental health professionals or clinicians to determine the presence of GAD and major depressive disorder (MDD) [12, 13]. The PHQ-4 is the most widely used self-reported screening instrument for anxiety and depression because of its ease of administration and demonstrated psychometric properties in general populations and patients [3, 6, 11, 13].

The psychometric properties of the PHQ-4 and its subscales have been examined among general adult populations in other countries and patients in clinical settings [6, 11–13, 16, 17]. These studies were conducted among Hispanic Americans [6], Colombians [17], Germans [13], Filipinos [16], patients in the U.S [5, 11, 12], and U.S. college students [2]. The results from these studies in the general population indicated strong internal consistencies of the PHQ-4 (*α* = 0.78–0.86), PHQ-2 (*α* = 0.75–0.80), and GAD-2 (*α* = 0.81–0.83). The authors also noted evidence of a two-factor structure or dimensionality of the PHQ-4 scale. The studies further revealed significant intercorrelations between the scales, and item-intercorrelations, supporting the construct validity of the measures.

Despite the usefulness of the PHQ-4 and its subscales (i.e., PHQ-2 and the GAD-2) for mass screening, little to no study examined the reliability and validity of these scales in the general U.S. adult population or based on U.S. nativity (i.e., foreign-born vs. the US-born). The reliability and validity results revealed in the previous studies cannot be generalized to all U.S. populations, given differences in populations’ experiences, culture, and socioeconomic status [18–20]. Most studies in the U.S. on anxiety and depression examined these disorders among the general population without considering differences between foreign-born individuals and US-born individuals [21]. A systematic review reported that foreign-born or migrant individuals experienced significant anxiety and depression [18]. Socioeconomic differences, migration stress, and difficulty adapting to their host countries’ culture significantly impact their mental health [18, 20]. In a study in the U.S., it was reported that anxiety and depression associated with US-nativity differed across ancestral origin groups, with those from Mexico, Eastern Europe, and Africa or the Caribbean having higher risks, especially foreign-born individuals who arrived at the age of 13 years or higher [22].

This current study, therefore, aimed to examine the reliability and validity of PHQ-4, PHQ-2, and GAD-2 in a large national sample of U.S. adults and based on their nativity. Specifically, we assessed the item characteristics, reliability, construct validity, and factorial structure of the PHQ-4, PHQ-2, and GAD-2. We also assessed the associations of the scale scores of PHQ-4, PHQ-2, and GAD-2 with the sociodemographic characteristics of U.S. adults to further examine the validity of these scales. Similarly, loneliness has been associated with mental health outcomes, including anxiety and depression [23]. Individuals experiencing loneliness are more burdened with anxiety or depression symptoms [23]. Hence, we evaluated the associations of the PHQ-4 scale and its subscales with loneliness to determine their validity in our sample. We expect to find a better-fit model for the two-factor structure of the PHQ-4 scale compared to the one-factor structure. We also expect to find unequal psychometric properties of the PHQ-4 scale based on U.S. nativity.

## Methods

### Study design and participants

The participants included a random sample of U.S. adults aged ≥ 18 years who were recruited in a national anonymous online cross-sectional survey. The survey participants’ recruitment and distribution, sponsored by the National Institute of Health, were executed by Qualtrics LLC between May 13, 2021, and January 9, 2022. The survey was developed and conducted in English. Qualtrics LLC oversampled low-income and rural individuals within US-born White, Black, Hispanic, and foreign-born populations to enhance the study participants’ representativeness. The survey was distributed to 10,000 participants, and 5,938 of them completed the survey, with 5,413 participants providing valid responses. The invalid responses included data we were unable to ascertain or incomplete surveys. We conducted a complete case analysis; therefore, 5,140 individuals with complete cases were included in the analysis. We assessed the differences in the sociodemographic characteristics of the complete cases and those excluded from the analysis; we found no significant differences in their sociodemographic characteristics. Besides, we had only 5% missingness, which is less than the 10% missingness threshold to result in bias estimates [24–26]. The Patient Health Questionnaire-4 (PHQ-4) scale was used to assess anxiety and depression among the participants. The survey also assessed the participants’ sociodemographic characteristics and loneliness. Ethical approval was obtained for the study on December 23, 2020, from the National Institutes of Health’s Institutional Review Board ([IRB] #000308).

### Measures

#### Main outcomes

The PHQ-4 is a 4-item unipolar self-reported scale comprising the PHQ-2 and the GAD-2 subscales [12, 13]. The PHQ-2 items are: (1) little interest or pleasure in doing things and (2) feeling down, depressed, or hopeless. The GAD-2 items are: (1) feeling nervous, anxious or on edge and (2) not being able to stop or control worrying. The items are based on how often the participants have been bothered in the last two weeks, and the response options include not at all = 0, several days = 1, more than half the days = 2, and nearly every day = 3. The total PHQ-2 and GAD-2 scores range from 0 to 6, and the PHQ-4 total score ranges from 0 to 12 [12, 13]. Total scores of ≥ 3 on any of the scales indicate anxiety (GAD-2), depression (PHQ-2), and both anxiety and depression (PHQ-4) symptoms.

#### Exposures

The 3-item UCLA Loneliness scale (short version) was used to measure loneliness among our survey participants [23, 27, 28]. The participants were asked to respond to the following three questions: (1) How often do you feel that you lack companionship? (2) How often do you feel left out? and (3) How often do you feel isolated from others? The response options for each question include 1 = hardly ever, 2 = some of the time, 3 = often). The total possible scores range from 3 to 9 [23, 27, 28]. The previous studies provided evidence of the reliability (alpha values ranged from 0.72 to 0.91) and validity (*r* = 0.82) of the 3-item UCLA Loneliness scale (short version) [23, 27, 28]. We found a similar alpha value of 0.88 for our study’s 3-item UCLA Loneliness scale.

Existing studies found that sociodemographic characteristics such as age, nativity, race/ethnicity, sexual and gender identity, level of education, marital status, employment, and income are known risk factors for anxiety and depression [6, 13, 22, 29]. Hence, we included these sociodemographic characteristics in our study to evaluate their associations with the PHQ-4 scale and its subscales of PHQ-2 and GAD-2.

### Statistical analysis

STATA/SE version 16 [30] and Mplus version 8.6 [31] were used to perform this study’s statistical analyses. STATA was used to conduct all the analyses, while both STATA and Mplus were used to conduct the one-factor and two-factor structure analyses. We analyzed the items’ frequency distributions and descriptive statistics for PHQ-4, PHQ-2, and GAD-2. We conducted summary statistics to determine each item’s means, standard deviations, skewness, and kurtosis. We used the skewness, kurtosis, quantile-quantile plot (Q-Q plot), and standardized normal probability or probability–probability plot (P-P plot) to examine the normality of the distributions. We also evaluated the items for missing data. Furthermore, we examined the internal consistencies of the PHQ-4, PHQ-2, and GAD-2 using Cronbach’s alpha (i.e., α) to determine their reliability [32, 33]. The alpha values of at least 0.70 are considered satisfactory or desirable [32–34]. Additionally, we computed the composite/construct reliability (also known as Jöreskog’s Rho) to test the composite reliability of the constructs [35, 36].

We examined the factorial validity and dimensionality of the PHQ-4 with confirmatory factor analysis (CFA). We evaluated the 2-dimensional structure (i.e., GAD-2 vs. PHQ-2) and a 1-dimensional structure (i.e., the PHQ-4 total score) of the PHQ-4 by examining two different factor models using the Maximum likelihood (ML) method, which is an effective and robust estimator in analysis involving large samples and normally distributed data [37]. We computed 95% confidence intervals (95% CIs) for the factor loadings. We assessed the two factors’ convergent and discriminant validities to evaluate their inter-correlation. Evidence of inter-correlation suggests convergent validity, while lack of evidence of or weak inter-correlation indicates discriminant validity [38–41]. We used average variance extracted (AVE) and squared correlations (SC) to determine the convergent and discriminant validities [38–41]. The AVE represents the average level of variance the latent constructs explain in their indicators relative to the total indicators’ variance or the amount of variance due to measurement error [38–41]. The AVE values greater than 0.50 (i.e., 50%) demonstrate evidence of convergent validity, further indicating that the latent construct explains more than 50% of the indicator variance [38–41]. There is evidence of discriminant validity when the AVE value is greater than or equal to the SC between the two latent constructs, further suggesting that the two latent constructs share more variance with their associated indicators than with their different sets of indicators in the model [38–41].

To examine the comparability of the factor structure of the PHQ-4 across native groups (US-born vs. foreign-born), we conducted a multiple-group confirmatory factor analysis (MCFA). We particularly evaluated the consistencies of the PHQ-4 scale for varying groups (i.e., US-born vs. foreign-born). Further, we examined and compared three increasingly restrictive models (i.e., configural, metric, and scale measurement invariance models) with the MCFA based on similar approaches used and recommended by other researchers [6, 29, 42].

We first examined *configural measurement invariance* by fitting a model (i.e., an unconstrained model) where all other parameters were freely estimated to determine whether the patterns of the factor loadings were the same in the two native groups or whether the model fits well equally in each of the two native groups. We then examined *metric measurement invariance* once the configural invariance was established. In this second model, factor loadings were constrained to be equal between the two groups. Once evidence of metric invariance was determined, the *scalar measurement invariance* (i.e., equal intercepts model) was examined by constraining the item intercepts and factor loadings. The *metric measurement invariance* model was compared with the *configural measurement invariance*, while the *scalar invariance* model was compared with the *metric measurement invariance* model. A non-significant test suggests the model under consideration fits the data just as well as the model estimated in the previous step of invariance testing.

Overall fit and model comparisons were evaluated using six criteria or indices. These indices include the Root Mean Square Error of Approximation (RMSEA), the Standardized Root Mean Residual (SRMR), the Comparative Fit Index (CFI), the Tucker-Lewis Index (TLI), and the likelihood ratio test (LRT). The RMSEA and SRMR values less than 0.08 suggest acceptable model fit, or values less than 0.05 indicate good model fit [6, 13]. Also, RMSEA values between 0.08 and 0.1 suggest marginal fits [6, 13, 43, 44]. The RMSEA was estimated at 95% CI. The CFI and TLI values greater than 0.95 indicate good model fit, while the values > 0.90 denote acceptable model fit [6, 13]. With the model comparisons, the LRT was used to compare a less restricted model (i.e., nested or simple model) to a more restricted model (i.e., complex or full model) with a statistically significant test suggesting a better fit of the more restricted model to the data than the less restricted model; otherwise, the more restricted model fits the data just as the less restricted model [45–49].

Analysis of variance (ANOVA) for at least three categories or groups and two-sample t-tests for two categories were used to assess the associations of sociodemographic characteristics with the PHQ-4 scale and its subscales of PHQ-2 and GAD-2. We performed the Bonferroni multiple-comparison test or Bonferroni adjustment for the ANOVA tests to account for multiple testing and determine which pairs of groups have significantly different scale scores. Additionally, we used Pearson’s correlation to assess the intercorrelations between the PHQ-4 scale, PHQ-2, and GAD-2 with the UCLA Loneliness scale - short version to determine the construct validity, specifically convergent validity. We computed the 95% CI for the Pearson’s correlation estimates.

## Results

### Descriptive statistics and sample characteristics

The sample characteristics can be found in Table 1. The majority of the participants were US-born individuals (77.26%, aged 35–49 (32.53%), females (62.63%), heterosexuals (89.22%), White Americans (43.00%), who had college or higher education (39.05%), currently employed (54.75%), married or living with a partner (54.28%), and had an annual household income of $75,000 or more (26.52%).

**Table 1:** Sample characteristics (*N* = 5,140)

	n (%)
**Nativity**	
US-born	3,971 (77.26)
Foreign-born	1,169 (22.74)
**Age**	
18–25	733 (14.26)
26–34	1,004 (19.53)
35–49	1,672 (32.53)
50–64	1,222 (23.77)
≥ 65	509 (9.90)
**Gender identity**	
Male	1,813 (35.27)
Female	3,219 (62.63)
Non-binary/transgender/other	108 (2.10)
**Sexual orientation**	
Heterosexual/straight	4,586 (89.22)
Lesbian/Gay	188 (3.66)
Bisexual	298 (5.80)
Other	68 (1.32)
**Race/ethnicity**	
White	2,210 (43.00)
Black/African American	1,255 (24.42)
Asian	531 (10.33)
Latino/Hispanic	934 (18.17)
Other	210 (4.09)
**Level of education completed**	
Less than High School	290 (5.64)
High School diploma or GED	1,175 (22.86)
Some college/vocational or technical school	1,668 (32.45)
College or higher degree	2,007 (39.05)
**Current employment**	
Employed	2,814 (54.75)
Unemployed	740 (14.40)
Unpaid/Voluntary/Apprenticeship	418 (8.13)
Permanently Sick/Disabled	289 (5.62)
In school or student	239 (4.65)
Retired	640 (12.45)
**Marital status**	
Never been married	1,568 (30.51)
Married/Living with a partner	2,790 (54.28)
Divorced/Widowed/Separated	782 (15.21)
**Annual household income**	
Less than $25,000	1,243 (24.18)
$25,000 to < $35,000	787 (15.31)
$35,000 to < $50,000	792 (15.41)
$50,000 to < $75,000	955 (18.58)
$75,000 or more	1,363 (26.52)

Table 2 shows the description of GAD2, PHQ4 and PHQ2, as well as the covariances and reliability. The mean (SD) scores of the PHQ-4, PHQ-2, and GAD-2 were 3.28 (3.67), 1.60 (1.89), and 1.67 (1.97), respectively. The mean scores for the items ranged from 0.80 to 0.85. Because we conducted a complete case analysis, the data had no missingness to compute the multiple or mean imputations [50, 51]. The descriptive statistics results in Table 2 showed that the skewness and kurtosis values were not extreme because the values for skewness were within ± 2, while the values for kurtosis were less than the|7.0| thresholds for high kurtosis [36]. Hence, the responses on the items do not deviate from normality, and the sample size is large enough to approximate normality and generate robust estimates [36, 52, 53]. The quantile-quantile plot (Q-Q plot) and standardized normal probability or probability–probability plot (P-P plot) also revealed the normality of the distributions (Figures not provided).

**Table 2:** Descriptive statistics for 5,140 participants on the GAD-2 and PHQ-2 items of the PHQ-4, and interitem correlations (covariances) and reliability

Item	n	Mean	SD	Skewness	Kurtosis	Item-rest or corrected item-total Correlation	Cronbach’s α	Composite reliability
**GAD-2 items (** ***n*** ** = 5,140)**								
Item1: Nervousness	5,140	0.85	1.03	0.94	2.63	0.83		
Item 2: Worries	5,140	0.82	1.04	0.99	2.67	0.83		
GAD-2 scale		1.67	1.97	0.96	2.70		0.90	0.90
**PHQ-2 items (** ***n*** ** = 5,140)**								
Item 3: Loss of interest	5,140	0.80	1.01	1.00	2.75	0.76		
Item 4: Depressive mood	5,140	0.80	1.01	1.02	2.81	0.84		
PHQ-2 scale		1.60	1.89	0.98	2.83		0.86	0.86
**Total scale score: PHQ-4 (** ***n*** ** = 5,140)**		3.28	3.67	0.95	2.78		0.92	0.92

Note: SD = standard deviation. α = Cronbach’s alpha

### Factorial validity and reliability

Given the normality of the responses, the correlations among the four items of PHQ-4 were examined using Pearson’s correlation (i.e., *r*) [36, 54, 55]. The correlation matrix displayed in Table 3 indicated statistically significant correlations among the four items (rs range from 0.68 to 0.82 and all *p* < 0.001). The correlation coefficients were higher than the ± 0.10 to 0.39 threshold for weak correlations [55]. The GAD-2 items (nervousness and worries: *r* = 0.82 [95% CI: 0.81, 0.83]) had a stronger correlation than the PHQ-2 items (loss of interest and depressive mood: *r* = 0.75 [95% CI: 0.74, 0.76]). As shown in Table 2, the internal consistencies were acceptable for the PHQ-4 scale (α = 0.92) and its subscales of PHQ-2 (α = 0.86) and GAD-2 (α = 0.90). The composite reliability values for the constructs were the same as the alpha values (Table 2).

**Table 3:** Correlation matrix between the four items assessing anxiety/depression scores (*N* = 5,140)

Measure/items	1	2	3
Item1: Nervousness	-		
Item 2: Worries	0.82*** (0.81, 0.83)	-	
Item 3: Loss of interest	0.69*** (0.67, 0.70)	0.68*** (0.66, 0.69)	-
Item 4: Depressive mood	0.75*** (0.74, 0.76)	0.77*** (0.76, 0.78)	0.75*** (0.74, 0.76)

*p* < 0.05*, *p* < 0.01**, *p* < 0.001***

### Dimensionality of PHQ-4: Two-factor model versus one-factor model

**Fig. 1:**
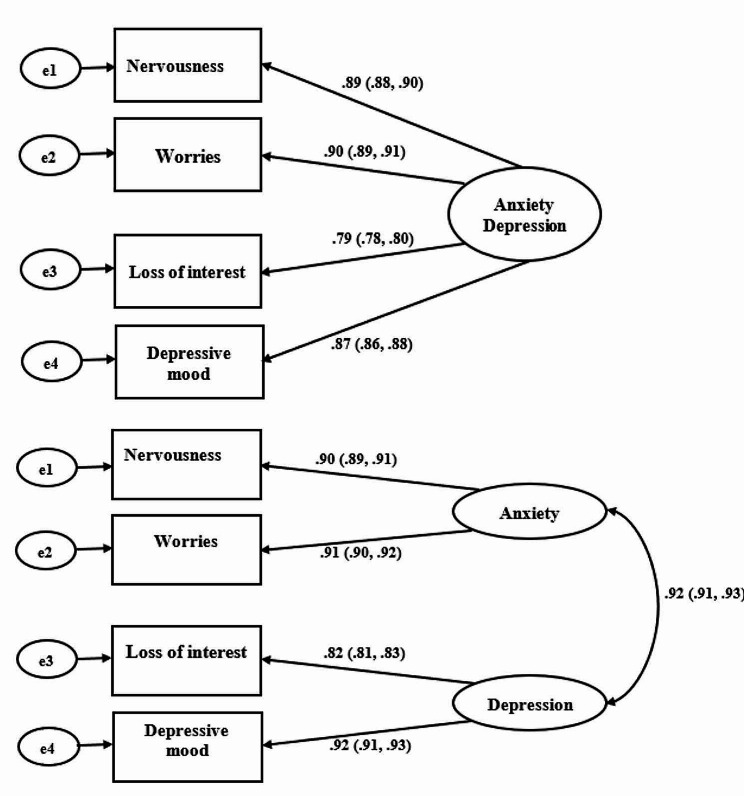
Confirmatory factor analyses’ estimates for the PHQ-4 one- and two-factor models

The comparison between one and two-factor structures of the PHQ-4 is displayed in Table 4. The results revealed strong evidence for two-factor structures of the PHQ-4. The model fit indices show that the two-factor model fits the data better than the one-factor model (two-factor model: RMSEA = 0.072; SRMR = 0.005; TLI = 0.990; CFI = 0.998 versus one-factor model: RMSEA = 0.203; SRMR = 0.025; TLI = 0.919; CFI = 0.973**)**. These results were further confirmed by the Likelihood Ratio Chi-square test for the model comparison (Likelihood Ratio Chi-Square Difference [**ΔLR χ**^**2**^] = 397.44, Δ*df* = 1, *p* < 0.001). As displayed in Fig. 1, the standardized factor loadings were higher for the PHQ-4 two-factor solution or dimension (*r*s = 0.82 to 0.92) than for the one-factor solution (*r*s = 0.79 to 0.90). Thus, the factor loadings on the PHQ-4 two-factor solution implied that 81% (i.e., 0.90^2^) and 82.81% (i.e., 0.91^2^) of the variances in items 1 and 2 are explained by GAD-2, respectively; 67.24% (i.e., 0.82^2^) and 84.64% (i.e., 0.92^2^) of the variances in items 3 and 4 are explained by PHQ-2, respectively. All the factor loadings were high and statistically significant (all *p* < 0.001). The two factors (i.e., anxiety and depression) were significantly correlated (*r* = 0.92). Thus, 84.64% (i.e., 0.92^2^) of the variance in GAD-2 and PHQ-2 is shared with the PHQ-4. We also evaluated the convergent and discriminant validities of the two factors. The AVE for GAD-2 (AVE = 0.821) and PHQ-2 (AVE = 0.755) were less than their SC (SC = 0.841), indicating no problem with convergent validity but a problem with discriminant validity. AVE for GAD-2 (AVE = 0.821) and PHQ-2 (AVE = 0.755) were greater than the 0.50 threshold, demonstrating evidence of convergent validity. The AVE values were, however, less than their SC value (SC = 0.841), indicating no evidence of discriminant validity. The composite reliability values (GAD-2: Jöreskog’s Rho = 0.90 and PHQ-2: Jöreskog’s Rho = 0.86) for the two factors were also higher than the AVE values for each factor, which supports the construct reliability.

**Table 4:** Goodness-of-fit indices for the PHQ-4 one-factor and two-factor models (*N* = 5,140)

Model	χ^2^	df	ΔLR χ^2^	Δdf	*p*	RMSEA (95% CI)	SRMR	TLI	CFI
Two-factor	27.635	1			< 0.001	0.072 (0.050, 0.096)	0.005	0.990	0.998
One-factor	425.077	2	397.442	1	< 0.001	0.203 (0.187, 0.219)	0.025	0.919	0.973

Note. **ΔLR χ**^**2**^ = Likelihood Ratio Chi-Square Difference. RMSEA = root mean square error of approximation; SRMR = standardized root mean square residual; TLI = Tucker Lewis Index; CFI = comparative fit index. 95% CI = 95% confidence interval

### Multiple group confirmatory factor analysis models

The results for the single-group CFA and the multiple-group CFA are shown in Table 5. The results of the single group CFA showed evidence of good model fit for both US-born and foreign-born groups, but the model appeared to have a better fit in the foreign-born group than the US-born group. The MCFA results indicated there was evidence that all the measurement invariance models fit the data well. The configural invariance model revealed similar factor structures in the two native groups. The metric invariance also showed that the factor loadings are equal across the two native groups. When the metric model was compared with the configural model, the test was not statistically significant (**ΔLR χ**^**2**^ = 3.49, Δ*df* = 3, *p* = 0.322), indicating a metric invariance. We proceeded to compute the scalar invariance model given the evidence of metric invariance. The scalar invariance model fits the data just as the metric invariance model (**ΔLR χ**^**2**^ = 1.34, Δ*df* = 3, *p* = 0.719); the assumption of equal item intercepts holds, and therefore, none of the two groups consistently have higher scores on the items than the other, adjusting for the latent construct. The practical goodness-of-fit indices (i.e., RMSEA, SRMR, TLI, and CFI**)** also confirmed the better-fitted models, or no worsening fits when applying the additional invariance constraints.

**Table 5:** Multigroup confirmatory factor analysis (MCFA) in two groups of US-born and foreign-born (*N* = 5,140)

Model	χ^2^	df	*p*	ΔLR χ^2^	Δdf		RMSEA (95% CI)	SRMR	TLI	CFI
**Single group solutions**										
US-born (*n* = 3,971)	409.237	2	< 0.001				0.226 (0.208, 0.245)	0.027	0.902	0.967
Foreign-born (*n* = 1,169)	40.351	2	< 0.001				0.128 (0.095, 0.164)	0.018	0.964	0.988
**Invariance models**										
Configural	449.587	4	< 0.001				0.208 (0. 192, 0. 225)	0.023	0.915	0.972
Metric	453.082	7	< 0.001	3.49^a^	3		0.157 (0. 145, 0. 170)	0.027	0.951	0.972
Scalar	454.426	10	< 0.001	1.34^b^	3		0.132 (0. 121, 0. 142)	0.027	0.966	0.972

Note. **ΔLR χ**^**2**^ = Likelihood Ratio Chi-Square Difference. RMSEA = root mean square error of approximation; SRMR = standardized root mean square residual; TLI = Tucker Lewis Index; CFI = comparative fit index. ^a^*p*= 0.322. ^b^*p*= 0.719

### Construct validity (Convergent validity)

The intercorrelation between the PHQ-4 total scale score and the UCLA Loneliness scale– short version was 0.63 (95% CI: 0.61, 0.65) (Table or Figure not provided). This correlation was 0.59 (95% CI: 0.57, 0.60) between the GAD-2 anxiety scale and the UCLA Loneliness scale– short version. The correlation between the PHQ-2 depression scale and the UCLA Loneliness scale– short version was 0.61 (95% CI: 0.59, 0.63). These intercorrelations were statistically significant (all *p* < 0.001), implying good convergent validity of the PHQ-4 scale and its subscales.

Table 6 presents the associations of the PHQ-4 scale and subscale scores with the sociodemographic characteristics of the general sample. This evaluation was to assess the validity of the constructs (PHQ-4 and its subscales) using the independent samples t-test and ANOVA to determine whether the scale scores vary or differ among the participants based on their sociodemographic characteristics. The PHQ-4 scale and its subscale scores were significantly associated with all the sociodemographic characteristics (all *p* < 0.001), suggesting differences in the scale scores between groups. The following interpretations of the mean scores are not based on p-values for the pairwise comparisons. US-born individuals had higher depression (i.e., PHQ-2), anxiety (i.e., GAD-2), and anxiety/depression scores than foreign-born individuals. The scores were also higher for younger adults between ages 18–25 or 26–34 years than for older adults; the scores for individuals who identified as non-binary, transgender, or other compared to those who identified as males or females; lesbian or gay and bisexual, especially bisexual, individuals exhibited higher scale scores than heterosexual/straight individuals. Latinos/Hispanics and other racial groups scored higher on the scales than Black/African, White, and Asian Americans; the participants with lower educational levels compared to those with higher educational levels had higher scale scores; these scores were higher for those currently unemployed, in unpaid work/voluntary job/apprenticeship, permanently sick or disabled, or students than for those who employed or retired. Participants who were never married scored higher on the scales than those who were divorced, widowed, separated, or married; the scores were higher for those who had less than $35,000 annual household income than for those who had income higher than $35,000.

**Table 6:** Association of PHQ-2, GAD-2 and PHQ-4 scores with demographic characteristics (*N* = 5,140)

	GAD-2, *n* = 5,140			PHQ-2, *n* = 5,140			PHQ-4, *n* = 5,140	
	Scale score	Group differences		Scale score	Group differences		Scale score	Group differences
	M (SD)	p-value		M (SD)	p-value		M (SD)	p-value
**Nativity**		< 0.001			< 0.001			< 0.001
US-born	1.74 (2.01)			1.65 (1.92)			3.39 (3.73)	
Foreign-born	1.45 (1.84)			1.43 (1.79)			2.89 (3.44)	
**Age**		< 0.001			< 0.001			< 0.001
18–25	2.32 (2.02)a			2.36 (1.92)			4.68 (3.68)	
26–34	2.16 (2.07)ab			2.00 (1.97)			4.16 (3.83)	
35–49	1.66 (1.97)			1.60 (1.88)			3.25 (3.66)	
50–64	1.37 (1.88)			1.27 (1.77)			2.64 (3.48)	
≥ 65	0.57 (1.14)			0.57 (1.23)			1.14 (2.21)	
**Gender identity**		< 0.001			< 0.001			< 0.001
Male	1.32 (1.83)			1.41 (1.83)			2.73 (3.48)	
Female	1.84 (2.02)			1.69 (1.91)			3.53 (3.73)	
Non-binary/transgender/other	2.55 (2.15)			2.36 (2.03)			4.91 (3.96)	
**Sexual orientation**		< 0.001			< 0.001			
Heterosexual/straight	1.56 (1.93)			1.49 (1.84)			3.05 (3.59)	
Lesbian/Gay	2.28 (2.03)a			2.17 (1.94)a			4.45 (3.75)a	
Bisexual	2.84 (2.12)b			2.75 (2.02)b			5.58 (3.86)b	
Other	2.69 (2.13)ac, bc			2.49 (1.99)ac, bc			5.18 (3.91)ac, bc	
**Race/ethnicity**		< 0.001			< 0.001			< 0.001
White	1.65 (2.03)a			1.50 (1.90)a			3.15 (3.76)a	
Black/African American	1.65 (1.90)ab			1.69 (1.86)ab			3.34 (3.55)ab	
Asian	1.35 (1.74)			1.41 (1.76)ac			2.76 (3.33)ac	
Latino/Hispanic	1.89 (2.02)bc			1.77 (1.88)bd			3.66 (3.69)bd	
Other	1.95 (2.11)ad, bd, cd			1.94 (2.12)be, de			3.89 (4.04)ae, be, de	
**Level of education completed**		< 0.001			< 0.001			< 0.001
Less than High School	2.27 (2.09)			2.29 (2.07)			4.56 (3.92)	
High School diploma or GED	1.89 (2.05)a			1.80 (1.93)a			3.69 (3.79)a	
Some college/vocational or technical school	1.82 (2.04)ab			1.70 (1.92)ab			3.52 (3.76)ab	
College or higher degree	1.34 (1.80)			1.31 (1.76)			2.65 (3.38)	
**Current employment**		< 0.001			< 0.001			< 0.001
Employed	1.60 (1.92)a			1.54 (1.84)a			3.14 (3.58)a	
Unemployed	2.15 (2.11)b			2.02 (2.01)b			4.17 (3.92)b	
Unpaid/Voluntary/Apprenticeship	1.77 (1.91)ac			1.57 (1.81)ac			3.35 (3.48)ac	
Permanently Sick/Disabled	2.37 (2.21)bd			2.34 (2.06)bd			4.71 (4.06)bd	
In school or student	2.43 (2.12)be, de			2.31 (1.96)be, de			4.74 (3.86)be, de	
Retired	0.80 (1.47)			0.81 (1.52)			1.61 (2.81)	
**Marital status**		< 0.001			< 0.001			
Never been married	1.99 (2.05)			1.97 (1.97)			3.96 (3.80)	
Married/Living with a partner	1.50 (1.92)a			1.39 (1.80)			2.88 (3.53)	
Divorced/Widowed/Separated	1.66 (1.95)ab			1.65 (1.92)			3.32 (3.70)	
**Annual household income**		< 0.001			< 0.001			< 0.001
Less than $25,000	2.00 (2.09)a			1.93 (1.97)a			3.93 (3.84)a	
$25,000 to < $35,000	2.03 (2.06)ab			1.91 (1.92)ab			3.94 (3.79)ab	
$35,000 to < $50,000	1.75 (1.94)c			1.71 (1.87)ac, bc			3.46 (3.60)bc	
$50,000 to < $75,000	1.62 (1.92)cd			1.50 (1.86)cd			3.11 (3.61)cd	
$75,000 or more	1.17 (1.75)			1.15 (1.72)			2.31 (3.30)	

^abcde^ Means differences in groups not statistically significant for the comparison of the scale scores between groups using the Bonferroni multiple-comparison test in the ANOVA tests. Thus, groups with letter combinations (e.g., ab, ac, cd) have no statistically significant mean differences. Groups without letter combinations (e.g., ab, ac, cd) have statistically significant mean differences

Differences in two groups were examined with a two-sample t-test, and the differences in at least three groups were evaluated using the ANOVA test

## Discussion

Previous studies examined the psychometric properties of the PHQ-4 scale among Hispanic Americans [6], Colombians [17], Germans [13], patients in the U.S [5, 11, 12], and U.S. college students [2]. However, our study was the first to evaluate the psychometric properties of the PHQ-4 scale among the general U.S. adult population and the comparison of the PHQ-4 scale across US-born and foreign-born Americans. Our findings provided evidence for the PHQ-4 scale, especially the two-factor structure of the PHQ-4, as a reliable and valid self-administered measure of anxiety and depression symptoms in the general U.S. adult population. The findings also demonstrated high internal consistency of the PHQ-4 scale (α = 0.92). The CFA results showed that the model fit the data well for both US-born and foreign-born groups, but the model fits the data better in the foreign-born group. Further examination of the factor invariance or consistency of the PHQ-4 scale for varying groups (US-born vs. foreign-born) using MCFA revealed that PHQ-4 could be used to assess anxiety and depression symptoms equally across US-born and foreign-born Americans, indicating that the scores on the PHQ-4 scale can be compared across these two native groups.

Consistent with previous findings [6, 13], our results showed evidence of the two-dimensional structure (i.e., GAD-2 vs. PHQ-2) compared to the one-dimensional structure (i.e., the PHQ-4 total score) of the PHQ-4 scale. These findings affirm the use of the PHQ-4 scale as a two-dimensional instrument to measure anxiety and depression symptoms in the general U.S. adult population. The reliabilities or the internal consistencies of the PHQ-4 scale (α = 0.92), PHQ-2 scale (α = 0.86), and GAD-2 scale (α = 0.90) in our study are higher than those reported in the general populations of Germany (PHQ-4: *α* = 0.82; PHQ-2: *α* = 0.78; and GAD-2: *α* = 0.75) and Colombia (PHQ-4: *α* = 0.86; PHQ-2: *α* = 0.83; and GAD-2: *α* = 0.79) by Löwe et al. [13] and Sanabria-Mazo et al. [17], respectively. The findings, therefore, indicate higher reliability of the PHQ-4 scale and its subscales in the general U.S. population compared to the general populations of Germany and Colombia. Existing studies indicated that anxiety and depression are strong co-occurring disorders with increased disability severity due to their comorbidity [1, 5, 6, 56]. Similarly, our findings revealed high intercorrelation of the subscales of PHQ-2 and GAD-2 (*r* = 0.92), which are higher than those reported in the German (*r* = 0.79) and Colombian (*r* = 0.68) studies. The two-dimensional factor loadings in our study (*r*s = 0.82 to 0.92) were also higher than those reported in the German (*r*s = 0.73 to 0.87) and the Colombian (*r*s = 0.71 to 0.92) studies. The high correlation between anxiety and depression in our study implies that they have a weak discriminant validity but strong convergent validity [12, 13]. Although these measures are conceptualized as different, their high correlation operates against their discriminant validity [12, 13]. Thus, the two measures did not share more variance with their associated indicators than with their different sets of indicators. Consequently, the two measures may not be distinguishable from each other; using only the PHQ-2 or GAD-2 to assess the symptoms of anxiety or depression may not fully assess the symptoms [12, 13]. Additional studies, especially longitudinal and clinical studies, are needed to further examine the stability of the discriminant and convergent validities, and the effectiveness of the two scales to screen for anxiety and depression symptoms in the general population.

It should, however, be noted that while our study was conducted among adults aged 18 years or more (*N* = 5,140) between May 2021 and January 2022, Sanabria-Mazo et al. [17] conducted their study among adults aged ≥ 18 years (*N* = 18,061) in Colombia between May and June 2020. Although these two studies were conducted during the COVID-19 pandemic, the Colombian study was conducted during the initial phase, while our study was conducted during the later phases of the pandemic. The higher reliabilities of the scales in our study could be due to the negative impact of the pandemic on the severity of anxiety and depression symptoms during the later phases of the pandemic, as mental health symptoms coupled with unemployment, social distancing stress, high rental costs, and inflation rates have worsened during the pandemic [1, 4, 57–59]. Thus, mental health symptoms increased during the pandemic and therefore, higher scores on the scale items could result in their higher inter-relatedness leading to higher reliability scores [60]. Similarly, Löwe et al. [13] also conducted their study among individuals aged ≥ 14 years (*N* = 5036) in Germany between May and June 2006, which reported lower reliabilities of the scales 16 years before the pandemic compared to those reported in our study and Colombia during the initial and later phases of the pandemic, respectively. Additionally, the differences in the internal consistencies could be attributed to younger samples in the German study compared to adult samples in our and Colombian studies.

Our results showed that the two-factor structure of the PHQ-4 was invariant across the two native groups (US-born vs. foreign-born individuals) in this study, although the model demonstrated better fit in the foreign-born group based on the model fit indices (RMSEA = 0.128, SRMR = 0.018, TLI = 0.964, and CFI = 0.988**)** than in the US-born group (RMSEA = 0.226, SRMR = 0.027, TLI = 0.902, and CFI = 0.967**)**. That is, the factor structure of the PHQ-4 is comparable or consistent across native groups, but the PHQ-4 scale might be used to screen anxiety and depression symptoms more accurately in the foreign-born population than in the US-born population. However, the RMSEA values for both nativity groups in the single-group analyses were higher than the 0.1 thresholds to suggest marginal fit. These high RMSEA values could be due to the use of ML estimator or method, which produces higher RMSEA values than using other estimators (e.g., maximum likelihood with robust standard errors [MLR], weighted least squares [WLS], weighted least squares mean adjusted [WLSM], weighted least squares mean-variance adjusted [WLSMV], unweighted least squares [ULS], and diagonally weighted least squares [DWLS]) [61–63]. Thus, RMSEA values are based on a fit function specific to an estimator [62]. RMSEA is a function of **χ**^**2**^ statistic, and ML estimator significantly influences **χ**^**2**^ test of model fit such as RMSEA fit index [62]. Nonetheless, we assessed other model fit indices that confirmed the better-fitted models; therefore, the high RMSEA values alone do not invalidate the adequacy of the CFA models.

Parallel with earlier studies [6, 13, 17, 22, 29], the findings of our study demonstrated the construct validity or convergent validity of the PHQ-4 and its subscales of PHQ-2 and GAD-2 in the general U.S. population. We found significant positive associations of the PHQ-4 scale and its subscales with the measure of loneliness (i.e., UCLA Loneliness scale - short version), denoting good convergent validity of the PHQ-4 scale and its subscales. The findings also imply that individuals who scored highly on the loneliness scale also scored highly on the PHQ-4 and its subscales. Therefore, loneliness might increase the risk of experiencing anxiety or depression symptoms, especially during the pandemic when social distancing rules were enforced, and social gatherings and traveling were restricted [23]. The scales were also associated with sociodemographic factors, including nativity, age, race/ethnicity, sexual and gender identity, level of education, marital status, employment, and income, as factors contributing to differences in anxiety and depression [6, 13, 22, 29]. Similar to previous studies [64–66], we observed that US-born individuals, younger adults aged 18–34 years, gender (i.e., non-binary/transgender/other) and sexual minority (i.e., lesbian or gay, bisexual, and others) individuals, Latinos/Hispanics and other racial groups (Black/African Americans and White Americans had similar scores), individuals with lower educational and income levels, unemployed individuals, and never been married individuals had higher anxiety (i.e., GAD-2), depression (i.e., PHQ-2), and their total scores (i.e., PHQ-4) compared with their counterparts. The evidence further suggests that the effect sizes of the PHQ-4 scores were larger than the scores of the subscales across all the sociodemographic factors. The higher effects sizes support the need to use the PHQ-4 scale other than the subscales to assess anxiety and depression symptoms as comorbid symptoms. Thus, individuals who experience anxiety also experience depression, and therefore, their comorbidity severity or scores can be higher than their individual scores, necessitating the use of the two-factor measure or PHQ-4 scale to assess anxiety and depression symptoms [1, 5, 6, 56].

Despite the strengths of this study in using large samples to evaluate the reliability and validity of the PHQ-4 scale and its subscales in the general U.S. adult population, the following limitations should be considered. First, because this is a cross-sectional study, we could not examine the stability of the measures among the same population over time. Next, the screening instruments (i.e., PHQ-4 and its subscales) were used to assess symptoms and not clinical diagnosis; we thus did not conduct clinical assessments to determine the presence or absence of clinical disorders (e.g., GAD and MDD) for clinical treatments. Moreover, because we performed an unweighted analysis, the generalizability of our findings is limited to the sample of the general population we analyzed. Furthermore, the self-reported responses could have resulted in misclassification or response biases, which often lead to underestimation of health behaviors and mental health, including symptoms of anxiety and depression. Finally, the lack of discriminant validity in our study may suggest limited evidence of the construct validity because only convergent validity was established despite the two measures being conceptualized as distinct [12, 13].

## Conclusions

To date, few studies have examined the reliability and validity of the PHQ-4 scale and its subscales in general populations. Notably, little to no studies examined these scales psychometric properties in the general U.S. population. Our study added evidence that the PHQ-4 is a reliable and valid instrument for assessing anxiety and depression symptoms in the general adult population. The two-factor structure of the PHQ-4 is a better and more efficient instrument than the one-factor structure, which further supports the evidence of the comorbidity of anxiety and depression symptoms and the need to use the two-factor measure [1, 5, 6, 56]. The two-factor structure was also consistent across US-born and foreign-born groups in this study, confirming this instrument as a potentially efficient rapid mass screener for anxiety and depression symptoms in the general U.S. population. Given limited public health resources (e.g., time, personnel, cost) for the assessment of anxiety and depression in clinical settings, widespread implementation of this instrument, especially during pandemics, can provide rapid information on anxiety and depression symptoms in the general population for consideration as these symptoms often affect health behaviors and decisions. Future studies may examine the test-retest reliability of the PHQ-4 scale and its subscales to determine their reliability or stability over time among the same general population to provide further evidence for the consistency of these measures in measuring anxiety and depression symptoms in the general population. Finally, other subgroup differences in the psychometric properties of the PHQ-4 and its subscales should be evaluated to determine their consistencies across major risk groups (e.g., sexual and gender minority, racial/ethnic minority individuals) as ongoing efforts to improve research to address health disparities.

## Data Availability

The data are available by making a request through Dr. FW per the new Data Management and Sharing Agreement plan.

## References

[1] Facts. & Statistics| Anxiety and Depression Association of America, ADAA. https://adaa.org/understanding-anxiety/facts-statistics. Accessed 17 Oct 2022.

[2] Khubchandani J, Brey R, Kotecki J, Kleinfelder JA, Anderson J (2016). The Psychometric properties of PHQ-4 depression and anxiety screening scale among College Students. Arch Psychiatr Nurs.

[3] Materu J, Kuringe E, Nyato D, Galishi A, Mwanamsangu A, Katebalila M (2020). The psychometric properties of PHQ-4 anxiety and depression screening scale among out of school adolescent girls and young women in Tanzania: a cross-sectional study. BMC Psychiatry.

[4] Twenge JM, Joiner TE (2020). U.S. Census Bureau-assessed prevalence of anxiety and depressive symptoms in 2019 and during the 2020 COVID-19 pandemic. Depress Anxiety.

[5] Kroenke K, Spitzer RL, Williams JBW, Löwe B (2009). An Ultra-brief Screening Scale for anxiety and depression: the PHQ–4. Psychosomatics.

[6] Mills SD, Fox RS, Pan TM, Malcarne VL, Roesch SC, Sadler GR (2015). Psychometric evaluation of the Patient Health Questionnaire–4 in hispanic americans. Hisp J Behav Sci.

[7] Koltai J, Raifman J, Bor J, McKee M, Stuckler D (2022). COVID-19 vaccination and Mental Health: a Difference-In-Difference analysis of the understanding America Study. Am J Prev Med.

[8] Sekizawa Y, Hashimoto S, Denda K, Ochi S, So M (2022). Association between COVID-19 vaccine hesitancy and generalized trust, depression, generalized anxiety, and fear of COVID-19. BMC Public Health.

[9] Smith K, Lambe S, Freeman D, Cipriani A (2021). COVID-19 vaccines, hesitancy and mental health. Evid Based Ment Health.

[10] Katon W, Roy-Byrne P (2007). Anxiety disorders: efficient screening is the first step in improving outcomes. Ann Intern Med.

[11] Kerper LF, Spies CD, Tillinger J, Wegscheider K, Salz A-L, Weiss-Gerlach E (2014). Screening for depression, anxiety, and general psychological distress in pre-operative surgical patients: a psychometric analysis of the Patient Health Questionnaire 4 (PHQ-4). Clinical Health Promotion - Research and best practice for patients. Staff Community.

[12] Kroenke K, Spitzer RL, Williams JBW, Löwe B (2010). The Patient Health Questionnaire somatic, anxiety, and depressive Symptom scales: a systematic review. Gen Hosp Psychiatry.

[13] Löwe B, Wahl I, Rose M, Spitzer C, Glaesmer H, Wingenfeld K (2010). A 4-item measure of depression and anxiety: validation and standardization of the Patient Health Questionnaire-4 (PHQ-4) in the general population. J Affect Disord.

[14] Siu AL, Bibbins-Domingo K, Grossman DC, Baumann LC, Davidson KW, Ebell M (2016). Screening for depression in adults: US Preventive Services Task Force Recommendation Statement. JAMA.

[15] Screening for Depression in Adults: U.S. Preventive Services Task Force Recommendation Statement. Ann Intern Med. 2009;151:784. 10.7326/0003-4819-151-11-200912010-00006 19949144

[16] Mendoza NB, Frondozo CE, Dizon JIWT, Buenconsejo JU (2022). The factor structure and measurement invariance of the PHQ-4 and the prevalence of depression and anxiety in a southeast Asian context amid the COVID-19 pandemic. Curr Psychol.

[17] Sanabria-Mazo JP, Useche-Aldana B, Ochoa PP, Rojas-Gualdrón DF, Mateo-Canedo C, Carmona-Cervelló M (2021). Social inequities in the impact of COVID-19 lockdown measures on the mental health of a large sample of the Colombian population (PSY-COVID study). J Clin Med.

[18] Bas-Sarmiento P, Saucedo-Moreno MJ, Fernández-Gutiérrez M, Poza-Méndez M (2017). Mental Health in immigrants Versus native Population: a systematic review of the literature. Arch Psychiatr Nurs.

[19] Peters BA, Yi SS, Beasley JM, Cobbs EN, Choi HS, Beggs DB (2020). US nativity and dietary acculturation impact the gut microbiome in a diverse US population. ISME J.

[20] Rivera B, Casal B, Cantarero D, Pascual CM. Adaptación de los servicios de salud a las características específicas y de utilización de los nuevos españoles. Informe SESPAS 2008. Gac Sanit. 2008;22 SUPPL. 1:86–95. 18405557

[21] Himle JA, Baser RE, Taylor RJ, Campbell RD, Jackson JS (2009). Anxiety disorders among African americans, blacks of Caribbean descent, and non-hispanic whites in the United States. J Anxiety Disord.

[22] Breslau J, Borges G, Hagar Y, Tancredi D, Gilman S (2009). Immigration to the USA and risk for mood and anxiety disorders: variation by origin and age at immigration. Psychol Med.

[23] Okruszek Ł, Aniszewska-Stańczuk A, Piejka A, Wiśniewska M, Żurek K. Safe but Lonely? Loneliness, anxiety, and depression symptoms and COVID-19. Front Psychol. 2020;11. 10.3389/fpsyg.2020.579181 PMC7747668 33343454

[24] Ayilara OF, Zhang L, Sajobi TT, Sawatzky R, Bohm E, Lix LM. Impact of missing data on bias and precision when estimating change in patient-reported outcomes from a clinical registry. Health Qual Life Outcomes. 2019;17. 10.1186/s12955-019-1181-2 PMC6585083 31221151

[25] Dong Y, Peng CYJ (2013). Principled missing data methods for researchers. Springerplus.

[26] Bennett DA (2001). How can I deal with missing data in my study?. Aust N Z J Public Health.

[27] Hughes ME, Waite LJ, Hawkley LC, Cacioppo JT (2004). A short scale for measuring loneliness in large surveys: results from two Population-Based studies. Res Aging.

[28] Russell DW (1996). UCLA Loneliness Scale (Version 3): reliability, validity, and factor structure. J Pers Assess.

[29] Gardner KJ, Qualter P (2011). Factor structure, measurement invariance and structural invariance of the MSCEIT V2.0. Pers Individ Dif.

[30] Why. Jul Stata| Stata. https://www.stata.com/why-use-stata/. Accessed 6 2021.

[31] Muthén LK, Muthén BO. Statistical Analysis With Latent Variables User’s Guide. 1998.

[32] Bland JM, Altman DG (1997). Statistics notes: Cronbach’s alpha. BMJ.

[33] Cronbach LJ (1951). Coefficient alpha and the internal structure of tests. Psychometrika 1951.

[34] Nunnally JC (1975). Psychometric theory’ 25 years ago and now. Educational Researcher.

[35] Raykov T. Estimation of Composite Reliability for Congeneric Measures. 10.1177/01466216970212006. 1997;21:173–84.

[36] Measurement Theory and Applications for the Social Sciences - Deborah L. Bandalos - Google Books. https://books.google.com/books?id=caxCDwAAQBAJ&printsec=copyright#v=onepage&q&f=false. Accessed 17 Oct 2022.

[37] Schermelleh-Engel K, Moosbrugger H, Müller H (2003). Evaluating the fit of structural equation models: tests of significance and descriptive goodness-of-fit measures. Methods Psychol Res Online.

[38] Alarcón D, Sánchez JA (2015). Assessing convergent and discriminant validity in the ADHD-R IV rating scale: user-written commands for average variance extracted (AVE), Composite Reliability (CR), and Heterotrait-Monotrait ratio of correlations (HTMT). Span STATA Meeting.

[39] Henseler J, Ringle CM, Sarstedt M (2015). A new criterion for assessing discriminant validity in variance-based structural equation modeling. J Acad Mark Sci.

[40] Fornell C, Larcker DF (1981). Evaluating Structural equation models with unobservable variables and measurement error. J Mark Res.

[41] Cheung GW, Cooper-Thomas HD, Lau RS, Wang LC. Reporting reliability, convergent and discriminant validity with structural equation modeling: a review and best-practice recommendations. Asia Pac J Manage. 2023;:1–39.

[42] Vandenberg RJ, Lance CE. A Review and Synthesis of the Measurement Invariance Literature: Suggestions, Practices, and Recommendations for Organizational Research. 10.1177/109442810031002. 2016;3:4–69.

[43] Kim H, Ku B, Kim JY, Park YJ, Park YB. Confirmatory and Exploratory Factor Analysis for Validating the Phlegm Pattern Questionnaire for Healthy Subjects. Evid Based Complement Alternat Med. 2016;2016. 10.1155/2016/2696019 PMC4804052 27051447

[44] Fabrigar LR, MacCallum RC, Wegener DT, Strahan EJ (1999). Evaluating the use of exploratory factor analysis in psychological research. Psychol Methods.

[45] Bryant FB, Satorra A. Principles and Practice of Scaled Difference Chi-Square Testing. 10.1080/107055112012687671. 2012;19:372–98.

[46] Satorra A, Bentler PM (2010). Ensuring positiveness of the scaled difference chi-square test statistic. Psychometrika.

[47] Alavi M, Visentin DC, Thapa DK, Hunt GE, Watson R, Cleary M (2020). Chi-square for model fit in confirmatory factor analysis. J Adv Nurs.

[48] He Y, Wang Z, Xu G. A Note on the Likelihood Ratio Test in High-Dimensional Exploratory Factor Analysis. 2021. 10.1007/s11336-021-09755-4 33770318

[49] Bai J, Duan J, Han X (2024). The likelihood ratio test for structural changes in factor models. J Econom.

[50] Lee KJ, Carlin JB (2010). Multiple imputation for Missing Data: fully conditional specification Versus Multivariate Normal Imputation. Am J Epidemiol.

[51] van Buuren S. Multiple imputation of discrete and continuous data by fully conditional specification. 10.1177/0962280206074463. 2016;16:219–42. 10.1177/0962280206074463 17621469

[52] Williams R, Allison PD, Moral-Benito E (2018). Linear dynamic panel-data estimation using maximum likelihood and structural equation modeling. Stata J.

[53] Williams R, Moral-Benito E, Allison PD. Dealing with non-normality in xtdpdml.

[54] Puth MT, Neuhäuser M, Ruxton GD (2014). Effective use of Pearson’s product–moment correlation coefficient. Anim Behav.

[55] Schober P, Schwarte LA (2018). Correlation coefficients: appropriate use and interpretation. Anesth Analg.

[56] Rodríguez-Muñoz M, de la Ruiz-Segovia F, Soto-Balbuena N, Le C, Olivares-Crespo HN, Izquierdo-Méndez ME (2020). The Psychometric properties of the Patient Health Questionnaire-4 for pregnant women. Int J Environ Res Public Health.

[57] McGinty EE, Presskreischer R, Han H, Barry CL (2020). Psychological distress and loneliness reported by US adults in 2018 and April 2020. JAMA.

[58] Coronavirus, Great Britain - Office for National Statistics. and anxiety,. https://www.ons.gov.uk/peoplepopulationandcommunity/wellbeing/articles/coronavirusandanxietygreatbritain/3april2020to10may2020. Accessed 27 Jan 2024.

[59] Leach CR, Rees-Punia E, Newton CC, Chantaprasopsuk S, Patel AV, Westmaas JL. Stressors and other pandemic-related predictors of prospective changes in psychological distress. Lancet Reg Health - Americas. 2021;4. 10.1016/j.lana.2021.100069 PMC8427739 34518825

[60] Tavakol M, Dennick R (2011). Making sense of Cronbach’s alpha. Int J Med Educ.

[61] Fischer R, Karl JA. A primer to (cross-cultural) multi-group invariance testing possibilities in R. Front Psychol. 2019;10 JULY:440108. 10.3389/fpsyg.2019.01507 PMC6657455 31379641

[62] Tarka P (PDF) The comparison of estimation methods on the parameter estimates and fit indices in SEM model under 7-point Likert scale, editor. Archives of Data Science. 2017;2:1–16.

[63] Xia Y, Yang Y, RMSEA, CFI (2019). TLI in structural equation modeling with ordered categorical data: the story they tell depends on the estimation methods. Behav Res Methods.

[64] Alegría M, Álvarez K, DiMarzio K (2017). Immigration and Mental Health. Curr Epidemiol Rep.

[65] McKnight-Eily LR, Okoro CA, Strine TW, Verlenden J, Hollis ND, Njai R (2021). Racial and ethnic disparities in the prevalence of stress and worry, Mental Health conditions, and increased substance use among adults during the COVID-19 pandemic — United States, April and May 2020. MMWR Morb Mortal Wkly Rep.

[66] Vahratian A, Blumberg SJ, Terlizzi EP, Schiller JS (2021). Symptoms of anxiety or depressive disorder and use of Mental Health Care among adults during the COVID-19 pandemic — United States, August 2020–February 2021. MMWR Morb Mortal Wkly Rep.

